# Epigallocatechin-3-Gallate Dampens Non-Alcoholic Fatty Liver by Modulating Liver Function, Lipid Profile, and Macrophage Polarization

**DOI:** 10.3390/nu13020599

**Published:** 2021-02-11

**Authors:** Yong Du, Laura Paglicawan, Sanam Soomro, Omar Abunofal, Sahar Baig, Kamala Vanarsa, John Hicks, Chandra Mohan

**Affiliations:** 1 Department of Biomedical Engineering, University of Houston, Houston, TX 77204, USA; laurapaglicawan@yahoo.com (L.P.); snmsoomro@gmail.com (S.S.); omar.nofal@hotmail.com (O.A.); sssbaig@gmail.com (S.B.); kvanarsa@Central.UH.EDU (K.V.); 2 Department of Pathology, Texas Children’s Hospital, Houston, TX 77030, USA; hicks@bcm.edu

**Keywords:** non-alcoholic fatty liver disease, epigallocatechin-3-gallate, inflammation, macrophage polarization

## Abstract

Epigallocatechin-3-gallate (EGCG) has been shown to attenuate obesity, fatty liver disease, hepatic inflammation and lipid profiles. Here, we validate the efficacy of EGCG in a murine model of non-alcoholic fatty liver disease (NAFLD) and extend the mechanistic insights. NAFLD was induced in mice by a high-fat diet (HFD) with 30% fructose. EGCG was administered at a low dose (25 mg/kg/day, EGCG-25) or high dose (50 mg/kg/day, EGCG-50) for 8 weeks. In HFD-fed mice, EGCG attenuated body and liver weight by ~22% and 47%, respectively, accompanied by ~47% reduction in hepatic triglyceride (TG) accumulation and ~38% reduction in serum cholesterol, resonating well with previous reports in the literature. In EGCG-treated mice, the hepatic steatosis score and the non-alcoholic steatohepatitis activity score were both reduced by ~50% and ~57%, respectively, accompanied by improvements in hepatic inflammation grade. Liver enzymes were improved ~2–3-fold following EGCG treatment, recapitulating previous reports. Hepatic flow cytometry demonstrated that EGCG-fed mice had lower Ly6C^+^, MHCII+ and higher CD206^+^, CD23^+^ hepatic macrophage infiltration, indicating that EGCG impactedM1/M2 macrophage polarization. Our study further validates the salubrious effects of EGCG on NAFLD and sheds light on a novel mechanistic contribution of EGCG, namely hepatic M1-to-M2 macrophage polarization. These findings offer further support for the use of EGCG in human NAFLD.

## 1. Introduction

Non-alcoholic fatty liver disease (NAFLD), the most common cause of liver diseases worldwide, is a condition in which excess fat builds up in the liver cells of patients who drink little or no alcohol. Although the exact prevalence of NAFLD is still unknown, it is estimated that its overall global prevalence diagnosed by imaging is around 25.24% (95% CI, 22.10–28.65) [1] In the US alone, this liver disorder affects an estimated 80 to 100 million people [2,3]. Moreover, it is not surprising that the prevalence of NAFLD is constantly increasing in the world, given the preponderance of a sedentary lifestyle and the pandemic spread of obesity.

The early-stage of NAFLD is a reversible condition characterized by steatosis of the liver, involving more than 5% of parenchyma with no evidence of hepatocyte injury [4,5]. Without proper control, the initial fat accumulation will trigger local inflammation and immune responses, followed by liver cell damage. The disease progresses to the 2nd stage of NAFLD, non-alcoholic steatohepatitis (NASH), with histopathological evidence of liver cell injury, placing patients at high risk of developing cirrhosis, hepatocellular carcinoma, and in some cases, the need for liver transplantation. NAFLD also has been considered as the hepatic manifestation of metabolic syndrome, being an independent risk factor for cardiovascular diseases and a significant cause of morbidity and mortality worldwide [5,6,7].

As a growing epidemic in humans, NAFLD is associated with a large economic burden on society and our healthcare system. Younossi and his colleagues have estimated that the direct annual medical costs of NAFLD are approximately $103 billion in the United States and € 35 billion a year in four developed European countries [8]. In a more recent study, the same group has explored the burden of NASH in the US and found that the lifetime costs of all NASH patients are around $222.6 billion [9]. These studies not only addressed the economic burden of NAFLD but also indicated the importance of treatment and management of this disease to reduce costs. Nevertheless, no ideal therapy exists to date. Lifestyle modification, especially weight loss, and pharmacologic therapy targeting dyslipidemia or insulin resistance remain the cornerstone of management [10,11]. While no pharmacologic therapy has been approved, our work herein examines the potential therapeutic efficacy of a natural compound, EGCG, on this disease.

EGCG, also known as epigallocatechin-3-gallate, is the most abundant and potent catechin in green tea. This compound is responsible for many of the beneficial effects of green tea that are reported in a variety of in vitro and in vivo studies [12]. Cumulative evidence from epidemiology studies, animal experiments, and clinical trials have revealed the multiple functions and therapeutic effects of EGCG. In aggregate, they demonstrate its antitumor, anti-inflammatory, antioxidant, as well as immunomodulatory effects. Moreover, EGCG also exhibits multiple tissue or organ-specific functions. Research conducted by Aktas and colleagues, for example, has shown that EGCG has neuroprotective and neurodegenerative effects, in which EGCG-fed experimental autoimmune encephalomyelitis (EAE) mice displayed significantly improved neuronal pathology [13,14,15]. Several in vitro and in vivo experiments have also demonstrated the anti-osteoclastogenic function of EGCG, assigning an important role to this natural compound in reducing bone erosion in rheumatoid arthritis (RA) [16,17,18].

Considering the multiple beneficial effects of EGCG and the very limited therapeutic options available for NAFLD, we examined the potential therapeutic effects of EGCG on NAFLD using a well-documented high-fat diet (HFD)-induced murine model. Our work showed that the administration of EGCG could lower body weight, protect liver function, and reduce liver fat accumulation and inflammatory cell infiltration that follow HFD feeding. In addition, in terms of mechanisms, our findings show that EGCG modulates serum lipid profile and liver macrophage differentiation and polarization.

## 2. Materials and Methods

### 2.1. Animal Care and Study Design

Male C57BL/6J mice (8 weeks old, bodyweight 22–30 g) were purchased from the Jackson Laboratory (Bar Harbor, ME, USA) and housed in a pathogen-free animal facility. The animal experiments were conducted in accordance with the Guide for the Care and Use of Laboratory Animals, and the animal use protocol was approved by the Institutional Animal Care and Use Committee at the University of Houston(EGCG/NAFLD: 16-038).

The NAFLD mouse model was induced using HFD (60 cal% fat (lard), 20 cal% carbohydrates and 20 cal% protein, purchased from Research Diets, Inc.) together with 30% fructose-containing drinking water for 16 weeks. These mice were randomly divided into three groups at the end of week 8: phosphate-buffered saline (PBS) control (*n* = 5); low EGCG group (EGCG-25; 25 mg/kg/day, *n* = 5); and high EGCG group (EGCG-50; 50 mg/kg/day, *n* = 5). One group of mice on a normal diet served as the unmanipulated healthy control (CTL, *n* = 5). EGCG (Sigma-Aldrich, St. Louis, MO, USA) was administered by oral gavage at a dose of 25 mg/kg or 50 mg/kg once per day for a total period of 8 weeks, starting at week 9 in the experiment.

### 2.2. Hepatic Triglyceride Determination and Lipid Staining

Mouse livers were homogenized in PBS. Tissue lipids were extracted with methanol/chloroform (1:2), dried in an evaporating centrifuge, and resuspended in 5% fat-free bovine serum albumin. Colorimetric assessment of triglyceride levels was carried out using a commercially available kit (Abcam, Cambridge, MA, USA). Values were normalized to protein concentration in liver homogenate using the Bradford assay (Bio-Rad Laboratories, Irvine, CA, USA). Frozen sections of the liver (10 µm) were stained with oil red O (Sigma, St. Louis, MO, USA) to determine hepatic lipid accumulation.

### 2.3. Liver Function and Serum Lipid Profile Measurement

Submandibular blood collection was performed using standard institutionally approved procedures to collect blood samples from each mouse once per month for liver function and serum lipid profile analysis. Aspartate aminotransferase (AST) and alanine aminotransferase (ALT) levels were measured using a HITACHI Clinical Analyzer 7070 (Hitachi High-Technologies Co., Tokyo, Japan).

### 2.4. Pathology Evaluation

Liver samples were collected for pathology evaluation at the end of the experiments. Simply, the liver was fixed using 10% formalin and embedded for H&E staining. Steatosis and liver inflammation were accessed using the following grading criteria: (1) Hepatic steatosis: grade 0: <5%, grade 1:5–33%, grade 2:33–66%, grade 3:66–100%; (2) Liver lobular inflammation: grade 0: None, grade 1: <2 foci per 200 × field, grade 2:2–4 foci per 200× field, grade 3: >4 foci per 200× field; (3) The non-alcoholic steatohepatitis (NAS) activity score is the total score of steatosis grade and lobular inflammation grade [19].

### 2.5. Intrahepatic Lymphocyte Isolation, Flow Cytometry and M1/M2 Differentiation

Intrahepatic lymphocytes were isolated following published protocols [20,21]. Briefly, the liver tissue was cut into small strips of ~1 mm^3^ pieces using a sterile blade in cold PBS and gently forced through a 75-µm cell strainer to remove cell clumps and large-size tissues. After centrifuging at 1500 rpm for 10 min, the cell pellet was resuspended in digestion buffer containing collagenase IV (500 mg/L) and DNase I (50 mg/L), then incubated at 37 C for 30 min. Next, 30 mL RPMI was added to the cell suspension, followed by incubation on ice for 5 min. The top 35 mL suspension was transferred into a new 50 mL tube and centrifuged at 1500 rpm, 4 c, for 10 min. The resulting pellet was then resuspended in 10 mL 35% Percoll and centrifuged at 1500 rpm for 30 min at room temperature. After RBC removal using erythrocyte lysis buffer, the cell suspension was ready for flow cytometry (FACS).

To immunophenotype the intrahepatic immune cells, antibodies directed against the cell surface markers were employed, including antibodies to CD45, F4/80, CD23, CD206, Ly6C, and MHC-II (BD PharMingen, San Diego, CA, USA). For M1/M2 subpopulation analysis, CD45^+^ cells were first selected from the live gate. Within the CD45^+^ cells, F4/80^+^ cells were gated and further characterized using antibodies to CD23^+^, CD206^+^, Ly6C^+^ and MHC-II^+^. The M1 subpopulation was either CD45^+^F4/80^+^Ly6C^+^ or CD45^+^F4/80^+^MHC-II^+^, while CD45^+^F4/80^+^CD206^+^ and CD45^+^F4/80^+^CD23^+^ groups were considered as M2 macrophages.

## 3. Statistical Analysis

All data are presented as mean ± SD. The difference between multiple groups was analyzed using one-way analysis of variance (ANOVA) followed by Tukey’s multiple comparison test. The difference between the two groups was analyzed using a 2-tailed *t*-test. A *p* value < 0.05 was considered statistically significant. All statistical analyses were performed using GraphPad Prism (V.6.0, GraphPad, San Diego, CA, USA).

## 4. Results

### 4.1. EGCG Administration Lowered Body Weight and Liver Weight of NAFLD Mice

**Figure 1:**
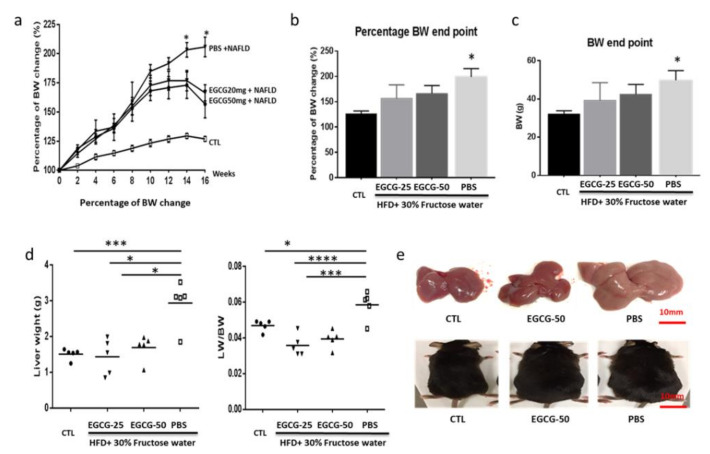
Epigallocatechin-3-gallate (EGCG) administration lowers body weight and liver weight in non-alcoholic fatty liver disease (NAFLD) mice. Diet-induced NAFLD mice were randomly divided into three treatment groups: phosphate-buffered saline (PBS), EGCG-25, and EGCG-50, and treated for 8 weeks. Body weight (BW) was recorded once per two weeks, and the percentage of BW change of each mouse was calculated compared to the baseline BW. (**a**) The percentage of BW change in each group. After high-fat diet (HFD) + 30% fructose feeding, BW of all mice increased constantly, but the percentage BW gain of EGCG-treated mice was lower than that in the PBS group (*n* = 5, * *p* < 0.05 compared to EGCG-treated groups at week 14 and week 16); (**b**) percentage BW change at study termination (*n* = 5, * *p* < 0.05 compared to EGCG-treated groups at week 16); (**c**) BW at the end of the experiment (*n* = 5, * *p* < 0.05 compared to EGCG-treated groups); (**d**) liver weight and liver weight-to-BW ratio among different treatment groups (*n* = 5, * *p* < 0.05; *** *p* < 0.01; **** *p* < 0.001); and (**e**) representative liver and mouse images from the unmanipulated (CTL), EGCG-50, and PBS groups.

Mice induced to have NAFLD were randomly divided into three groups: PBS, EGCG-25, and EGCG-50. All groups were continuously fed with HFD and 30% fructose during the entire period of the experiment, over a total of 16 weeks. As Figure 1,b show, the percentages of body weight gain in the EGCG-25 and EGCG-50 groups were significantly lower than that of the PBS group (*p* < 0.01, compared to EGCG-25, *p* < 0.05 compared to EGCG-50, respectively, one-way ANOVA test). At the end of the experiment, the mean body weights of the EGCG-25 and EGCG-50 mice were 39.37 ± 4.08 g and 42.6 ± 2.26 g, respectively, while it was 51.9 ± 2.26 g in the PBS group (Figure 1c, *p* < 0.05, compared to EGCG-25 and EGCG-50 mice, 2-tailed *t*-test).

Similarly, both the liver weight and the ratio of liver weight to the body weight of EGCG-treated groups were dramatically lower than those of the PBS group. As shown in Figure 1d, the mean liver weights in the EGCG-25 and EGCG-50 groups were 1.44 ± 0.50 g, and 1.69 ± 0.36 g, respectively, while that in the PBS group was 2.94 ± 0.63 g (compared to both the EGCG-25 and EGCG-50 groups, *p* < 0.001, one-way ANOVA test).

### 4.2. EGCG Reduced Liver Fat Accumulation

**Figure 2:**
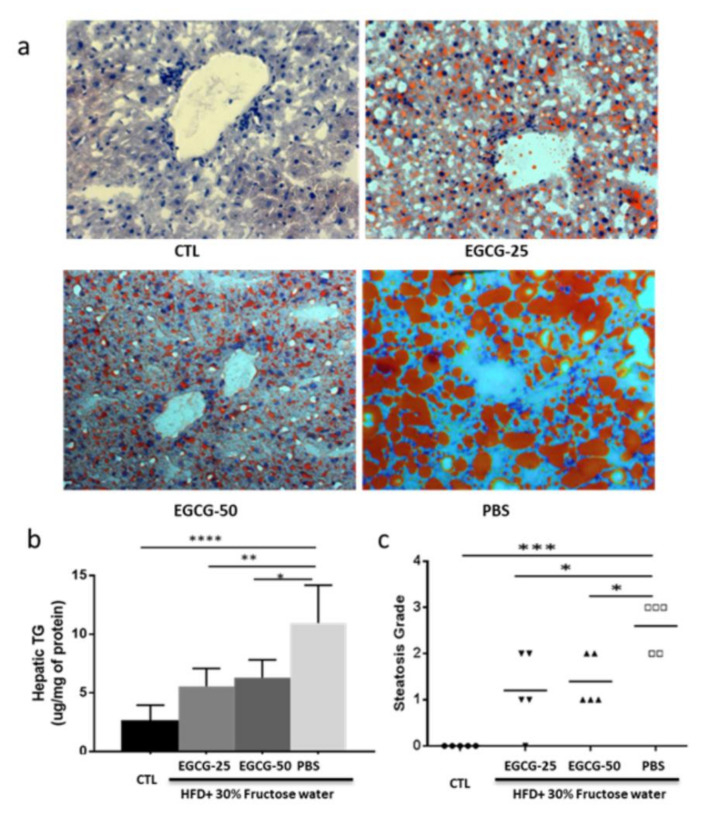
EGCG reduced liver fat accumulation in the treated groups. Diet-induced NAFLD mice were randomly divided into three treatment groups: PBS, EGCG-25, and EGCG-50, and treated for 8 weeks. Shown data were obtained at the end of the study. (**a**) Representative oil red staining image from each group is shown; (**b**) hepatic TG level of each group. Liver tissues were homogenized for TG level measurement, and data are presented as μg/mg of protein. The PBS group exhibited significantly higher hepatic TG levels, compared to the unmanipulated healthy control (CTL) (*n* = 5, **** *p* < 0.001), EGCG-25 (*n* = 5, ** *p* < 0.01), and EGCG 50 groups (*n* = 5, * *p* < 0.05 (one-way ANOVA test); and (**c**) steatosis score in each group. PBS group exhibited significantly higher steatosis score, compared to the unmanipulated healthy control (CTL) (*n* = 5, *** *p* < 0.01), EGCG-25 (*n* = 5, * *p* < 0.05), and EGCG 50 groups (*n* = 5, * *p* < 0.05 (one-way ANOVA test).

The level of hepatic TG in the EGCG-treated mice (EGCG-25:5.55 ± 1.53; EGCG-50:6.30 ± 1.53) was significantly lower than that in the PBS group (10.96 ± 3.23; *p* < 0.01, vs. EGCG-25; *p* < 0.05, vs. EGCG-50 group) by one-way ANOVA test, as shown in Figure 2b. Oil red staining allowed us to assess the level of fat deposition and the severity of liver damage, which indicated reduced fat accumulation in EGCG-treated mice (Figure 2a).

**Figure 3:**
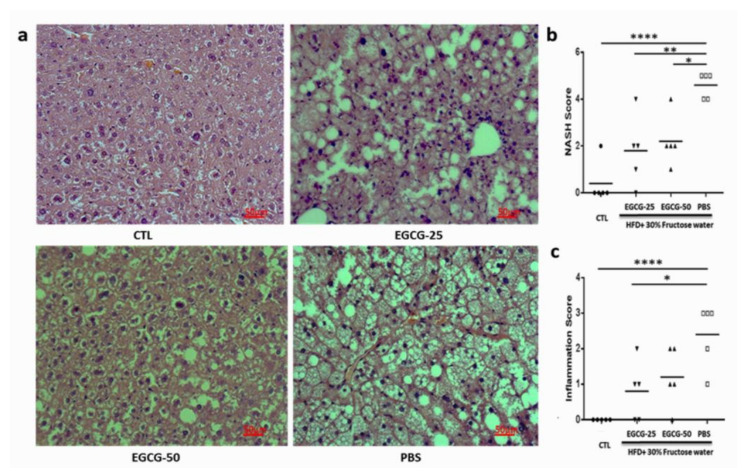
EGCG prevented pathological liver damage in NAFLD mice. Diet-induced NAFLD mice were randomly divided into three treatment groups: PBS, EGCG-25, and EGCG-50, and treated for 8 weeks. Shown data were obtained at the end of the study. Liver tissues were collected for pathology evaluation, including liver steatosis, liver inflammation, and hepatocyte injury. (**a**) Representative H&E staining image of each group; (**b**) NAS score; (**c**) inflammation score; (*n* = 5, * *p* < 0.05, ** *p* < 0.01; **** *p* < 0.001, as indicated in figure.

The liver steatosis score reflects the degree of fat accumulation in the liver. The average liver steatosis scores were 1.2 ± 0.84 and 1.4 ± 0.55 in the EGCG-25 and EGCG-50 groups, respectively, while the score was 2.6 ± 0.55 in the PBS group (*p* < 0.01 vs. EGCG-25 and *p* < 0.01 vs. EGCG-50, one-way ANOVA test; Figure 3). Taking these data together, EGCG treatment dramatically reduces liver fat deposition, even when these mice were continually fed with HFD and fructose-drinking water.

### 4.3. EGCG Prevented Pathological Liver Damage in NAFLD Mice

In addition to the reduced body weight and decreased hepatic accumulation, EGCG administration improved liver pathology. Figure 3a shows representative H&E images of each group. The NAS activity score represents the overall degree of liver steatosis, liver inflammation, and hepatocyte injury. These scores were 1.80 ± 1.4 and 2.2 ± 1.01 in the EGCG-25 and EGCG- 50 groups, respectively, significantly lower than that in the PBS group (4.6 ± 0.55; Figure 3b, *p* < 0.01 vs. EGCG-25 group and *p* < 0.05 vs. EGCG-50 group; one-way ANVOA test). Similarly, the inflammation grade (Figure 3c) was also significantly lower than those in the PBS group. Together, these findings indicate that EGCG administration ameliorates liver pathology.

### 4.4. EGCG Treatment Improved Liver Dysfunction in NAFLD Mice

**Figure 4:**
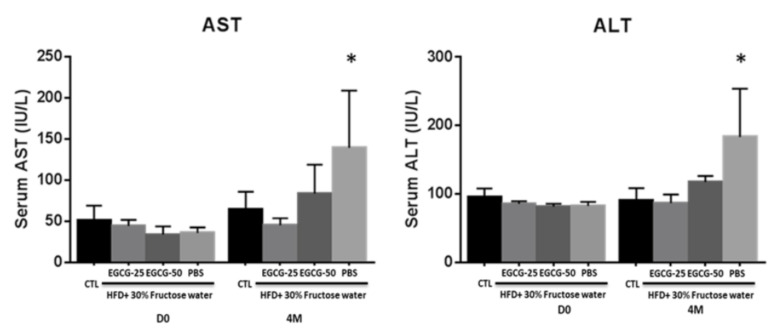
EGCG treatment improved liver dysfunction in NAFLD mice. Diet-induced NAFLD mice were randomly divided into three treatment groups: PBS, EGCG-25, and EGCG-50, and treated for 8 weeks. Plotted are AST and ALT levels before and after EGCG treatment. After HFD + 30% fructose feeding, the PBS groups exhibited increased aspartate aminotransferase (AST) and alanine aminotransferase (ALT) levels by 2–3-fold indicating liver dysfunction. After EGCG treatment, both EGCG-25 and EGCG-50 groups exhibited improved liver function (*n* = 5, * *p* < 0.05, PBS group compared to other groups).

In our study, both AST and ALT levels increased by ~2–3-fold in the PBS group at the end of the experiment (mean AST = 133.2 U/L and ALT = 183.4 U/L), in comparison to the baseline (mean AST = 35.62 U/L and ALT = 82.80 U/L). After EGCG treatment, liver dysfunction was significantly improved in both the EGCG-25 (AST 44.8 ± 8.85 U/L, ALT 86.8 ± 12.72 U/L, respectively, *p* < 0.001) and EGCG-50 groups (AST± 15.13 U/L, ALT 117.6 ± 9.13 U/L, respectively, *p* < 0.05), as displayed in Figure 4.

### 4.5. EGCG Treatment Improved Lipid Profiles in HFD Fed Mice

**Figure 5:**
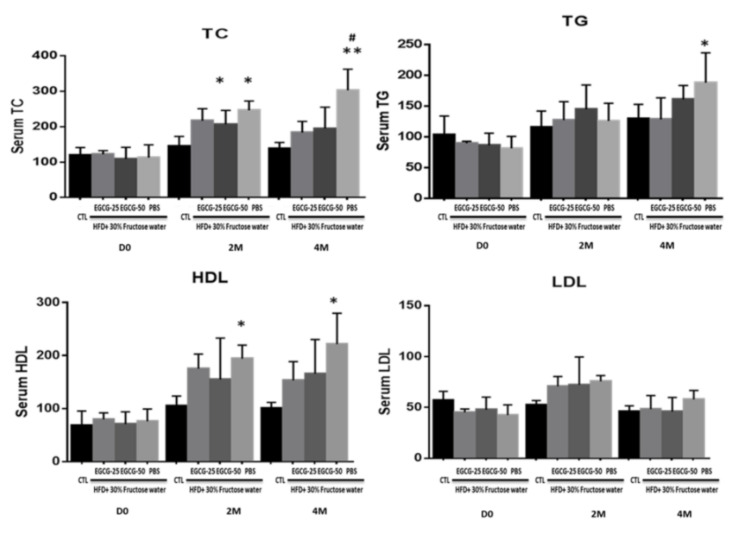
EGCG treatment improved the lipid profile in NAFLD mice. Diet-induced NAFLD mice were randomly divided into three treatment groups: PBS, EGCG-25, and EGCG-50, and treated for 8 weeks. Mice were examined 4 months following commencement of treatment. Hepatic triglyceride (TG) and high-density lipoprotein (HDL) levels in the EGCG-treated group was not altered compared with pretreatment levels. The TC level of the PBS group was significantly higher than that of the CTL (* *p* < 0.05, ** *p* < 0.01 vs. CTL of the same group/time-point; and #, *p* < 0.01 vs. EGCG-25 and EGCG 50 at the same group/time point). However, no differences were detected in the levels of low-density lipoprotein ( LDL).

After the HFD diet, as expected, the NAFLD mice exhibited increased total cholesterol (TC), TG, and high-density lipoprotein (HDL) levels compared to the non-HFD control group. After a 2-month EGCG treatment, the TC level of the EGCG-50 group reduced to 183.9 ± 31.42 mg/dL, while it was 195.5 ± 59.79 mg/dL in the EGCG-25 group, and both were significantly lower than that in the PBS group (303.5 ± 59.1342 mg/dL, *p* < 0.01; Figure 5). Similarly, HDL levels of EGCG-25 and EGCG-50 groups were lower than those in the PBS group (*p* < 0.05). TG levels in the EGCG-treated mice remained stable in comparison to pretreatment levels and were comparable to that in the PBS group.

### 4.6. EGCG Treatment Reduced Liver Macrophage Infiltration and Modulated M1/M2 Polarization in NAFLD Mice

**Figure 6:**
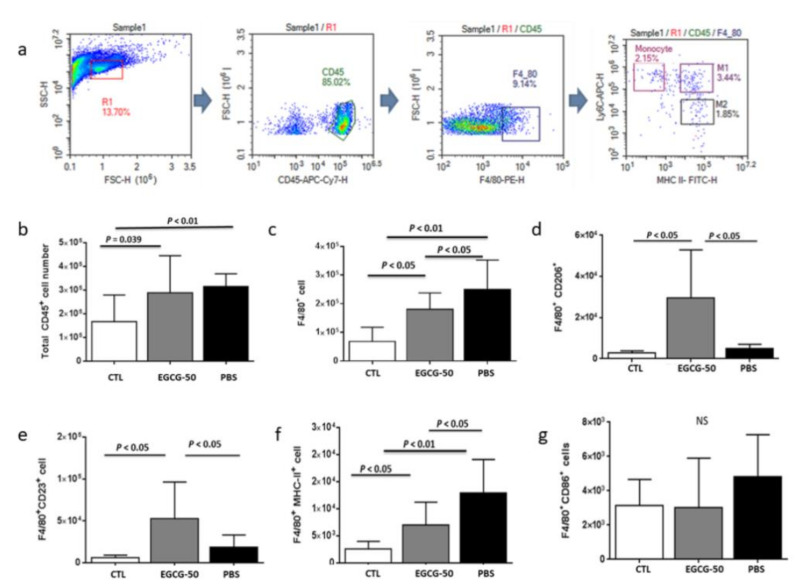
EGCG treatment reduced liver macrophage infiltration and modulated M1/M2 polarization in NAFLD mice. Diet-induced NAFLD mice were randomly divided into three treatment groups: PBS, EGCG-25, and EGCG-50, and treated for 8 weeks. Intrahepatic macrophages were immunophenotyped using flow cytometry. The M1 subpopulation was defined as either CD45^+^F4/80^+^Ly6C^+^ or CD45^+^F4/80^+^MHC-II^+^, while CD45^+^F4/80^+^CD206^+^ and CD45^+^F4/80^+^CD23^+^ cells were considered as M2 macrophages. (**a**) The sequential gating strategy used for intrahepatic immune cell analysis; (**b**) total CD45^+^ cells; (**c**) CD45^+^F4/80^+^ cells; (**d**) CD45^+^F4/80^+^CD206 M2 cells; (**e**) CD45^+^F4/80^+^CD23^+^ M2 cells; (**f**) CD45^+^F4/80^+^MHC-II^+^ M1 cells; and (**g**) CD45^+^F4/80^+^Ly6C^+^ M1 cells. All cell numbers shown are the number of cells per mouse liver.

NAFLD mice (both PBS and EGCG-50 groups) showed increased numbers of intra-hepatic CD45^+^ cells (leukocytes) compared to non-NAFLD mice (Figure 6a,b), indicating enhanced inflammatory infiltration after NAFLD induction. Compared to the PBS group, EGCG treatment reduced the numbers of infiltrating CD45^+^F4/80^+^ macrophages (Figure 6c). Importantly, EGCG treatment increased both CD45^+^F4/80^+^CD206^+^ and CD45^+^F4/80^+^CD23^+^ cells and lowered the number of CD45+F4/80+MHC-II+ infiltrating cells, implicating a potential role of EGCG in inducing M1-to-M2 polarization (Figure 6d–g).

## 5. Discussion

Non-alcoholic fatty liver disease (NAFLD) is the most common liver disease representing a growing epidemic in humans. It is estimated that the global prevalence of NAFLD is around 25.2% [1] and that at least 80 to 100 million adults suffer from this disease in USA alone [2,3]. More importantly, with the pandemic spread of obesity, this disease will dominate as the leading cause of end-stage liver disease in the coming decades. EGCG is a natural compound with various salubrious effects. In this study, we aimed to examine the potential therapeutic efficacy of EGCG in NAFLD. Our work indicates that EGCG treatment could lower body weight, liver weight, and fat accumulation. In parallel, the administration of EGCG improved liver function, serum lipid profile, and liver pathologic changes.

Obesity and overweight constitute the second leading cause of preventable death in the United States. They also represent the most common symptoms and the early signs associated with a diagnosis of NAFLD [22,23]. Several clinical epidemiology studies have proven that losing 3% to 6% of body weight could reduce liver fat levels by 35% to 40% [23], and losing more than 7% is associated with NAFLD remission [22]. Therefore, patients with NAFLD should benefit from any intervention that can safely lower body weight. Emerging evidence from various epidemiologic studies has shown the benefits of EGCG on body fat and body weight reduction as well as on body weight maintenance [24,25,26]. Our results show that both low- and high-dose treatments using EGCG could significantly reduce mouse body weight. As Figure 1a–c shows, EGCG treatment for four weeks was enough to prevent the mouse from body weight gain. At the end of the experiment, the mean body weights of EGCG-25 and EGCG-50 mice were 39.37 ± 4.08 g and 42.59 ± 2.26 g, respectively, while that of the PBS group was 51.9 ± 2.26 g (*p* < 0.05, *n* = 5, 2-tailed *t*-test). Similarly, the liver weight and the liver/body weight ratio of the EGCG-25 and EGCG-50 groups were dramatically decreased in comparison to the PBS group. Indeed, the beneficial impact of EGCG on body weight and liver weight in NAFLD has been observed in previous studies of EGCG treatment of murine NAFLD [27,28,29].

Consistent with the body weight loss, liver fat levels in the EGCG-25 and EGCG-50 groups were significantly reduced, as evidenced by oil red staining (Figure 2a), hepatic triglyceride determination (Figure 2b), and liver steatosis grading (Figure 2c). The lipid-modulating effect of EGCG has been reported in various studies [29,30,31,32]. In HFD-fed rats, four weeks of EGCG treatment significantly lowered serum TC and LDL levels [30], while decreased liver lipid has also been observed in EGCG-treated-chronic ethanol-fed rats [31]. In a small-scale clinical trial, EGCG was able to decrease serum TC, TG, and HDL [32]. Taking these together, it can be concluded that EGCG modulates lipid metabolism at both systemic and local tissue levels.

As mentioned above, the excess fat accumulation associated with NAFLD progression has been documented to trigger local inflammation, scar formation and fibrosis, eventually leading to liver dysfunction. EGCG treatment was able to prevent NAFLD mice from liver dysfunction. As shown in Figure 3, after HFD/fructose feeding, the PBS group showed increased AST and ALT levels, as compared to the EGCG-25 and EGCG-50 groups. In this study, we applied the alcoholic steatohepatitis (NAS) activity score to evaluate the severity of the disease [19]. After EGCG treatment, the NAS score of the EGCG-25 and EGCG-50 groups were 1.80 ± 1.4 and 2.2 ± 1.0, respectively, which were lower than the score of the PBS group (4.6 ± 0.55; *p* < 0.05; 2-tailed *t*-test). In addition, EGCG treatment also reduced the inflammation score, as well as the steatosis grade, with a significant statistical difference when compared to the PBS group, again recapitulating previous observations in the literature [29,33].

Hepatic macrophages account for the largest nonparenchymal cell population in the liver. These cells play a central role in the pathogenesis of liver inflammation and fibrosis. Infiltrating liver macrophages can be classified into M1 and M2 types, with different immune responses and immunomodulatory functions [34]. EGCG has been reported to polarize M1 to M2 cells. In a spinal cord injury model, EGCG administration modulated the expression of different macrophage markers, macrophage phenotypes and associated inflammatory responses [35]. Similarly, in a murine LPS-induced acute lung injury model, EGCG treatment modulated macrophage polarization towards M2 status, as evidenced by reduced proinflammatory M1 mediator expression and increased M2 marker expression [36]. Chu and colleagues also reported that EGCG-modified collagen promoted M2 macrophage recruitment reduced the secretion of inflammatory mediators, and favored M2 polarization [37,38,39]. In agreement with these reports, our work showed that the EGCG-treated group had decreased M1 but increased M2 macrophages, supporting the potential effect of EGCG on M1/M2 polarization. The increased M2 macrophages in EGCG-treated mice may be elaborating various immunomodulatory cytokines in order to reduce hepatic inflammation, although this needs to be formally tested.

To place our studies in the context of previous work done in this field, we compared our findings with those reported in the literature [27,28,29,31,33,40,41,42,43,44,45,46]. There is ample evidence in the literature that EGCG attenuates obesity [28,40], insulin resistance [33] and fatty liver disease [41] in rodents fed a high-fat diet by increasing activity of mitochondrial respiratory chain complexes [42], suppressing hepatic cholesterol synthesis [43], decreasing bile acid and lipid absorption [44], and modulating the interaction between gut microbiota and bile acids [45,46]. In addition to validating previous reports in the field, the current study makes a novel contribution in terms of demonstrating the role of EGCG in facilitating M1-to-M2 intra-hepatic macrophage polarization, an observation that merits further mechanistic dissection.

## Data Availability

Please refer to suggested Data Availability Statements in the section “MDPI Research Data Policies” at https://www.mdpi.com/ethics.

## Abbreviations

EGCG	epigallocatechin-3-gallate
NAFLD	eon-alcoholic fatty liver disease
HFD	high-fat diet
TG	triglycerides
HDL	high-density lipoprotein
NASH	non-alcoholic steatohepatitis
EAE	experimental autoimmune encephalomyelitis
AST	aspartate aminotransferase
ALT	alanine aminotransferase
NAS	non-alcoholic steatohepatitis
LDL	low-density lipoprotein
TC	total cholesterol
